# No beneficial use of the wearable cardioverter defibrillator among patients suffering from inherited and congenital heart disease: data from a European multicenter registry

**DOI:** 10.3389/fcvm.2024.1384736

**Published:** 2024-07-10

**Authors:** Katharina Koepsel, Tobias C. Dreher, Christian Blockhaus, Michael Gotzmann, Norbert Klein, Thomas Kuntz, Dong-In Shin, Hendrik Lapp, Fabian Schiedat, Mohammad Abumayyaleh, Thomas Beiert, Christian Weth, Boldizsar Kovacs, Stephanie Rosenkaimer, Jacqueline Kowitz, Ardan Muammer Saguner, Julia W. Erath, Firat Duru, Andreas Mügge, Ibrahim Akin, Assem Aweimer, Nazha Hamdani, Ibrahim El-Battrawy

**Affiliations:** ^1^ Department of Cellular and Translational Physiology and Institute für Forschung und Lehre (IFL), Institute of Physiology, Molecular and Experimental Cardiology, Ruhr-University Bochum, Bochum, Germany; ^2^ Department of Cardiology and Angiology, Bergmannsheil University Hospital, Ruhr University of Bochum, Bochum, Germany; ^3^ Department of Cardiology, Angiology, Haemostaseology and Medical Intensive Care, University Medical Center Mannheim, Medical Faculty Mannheim, Heidelberg University, Mannheim, Germany; ^4^ Department of Cardiology, Heart Centre Niederrhein, Helios Clinic Krefeld, Krefeld, Germany; ^5^ Faculty of Health, School of Medicine, University Witten/Herdecke, Witten, Germany; ^6^ Department of Cardiology and Rhythmology, University Hospital St. Josef-Hospital Bochum, Cardiology and Rhythmology, Ruhr University Bochum, Bochum, Germany; ^7^ Department of Cardiology, Angiology and Internal Intensive-Care Medicine, Klinikum St. Georg GGmbH Leipzig, Leipzig, Germany; ^8^ Department of Internal Medicine II, Heart Center Bonn, University Hospital Bonn, Bonn, German; ^9^ Department of Cardiology, Marienhospital Gelsenkirchen, Gelsenkirchen, Germany; ^10^ Department of Cardiology and Angiology, Clinic Saarbrücken GGmbH, Saarbrücken, Germany; ^11^ Department of Cardiology, University Heart Center, University Hospital Zurich, Zurich, Switzerland; ^12^ Department of Cardiology, Frankfurt University Hospital, Goethe University, Frankfurt am Main, Germany; ^13^ HCEMM-Cardiovascular Research Group, Department of Pharmacology and Pharmacotherapy, University of Budapest, Budapest, Hungary; ^14^ Department of Physiology, Cardiovascular Research Institute Maastricht, University Maastricht, Maastricht, Netherlands

**Keywords:** inherited channelopathies, sudden cardiac death, wearable-cardioverter defibrillator, congenital heart diasease, ventricular arrhythmia

## Abstract

**Background:**

Data on the use of the wearable cardioverter defibrillator in patients suffering from inherited and congenital heart disease are limited. Consequently, evidence for guideline recommendations in this patient population is lacking.

**Methods:**

In total 1,675 patients were included in a multicenter registry of eight European centers. In the present cohort, we included 18 patients suffering from congenital and inherited heart disease.

**Results:**

Nine patients (50%) were male with a mean age of 41.3 ± 16.4 years. Four patients suffered from hypertrophic cardiomyopathy (HCM), four patients suffered from non-compaction cardiomyopathy (NCCM), two patients were diagnosed with arrhythmogenic right ventricular cardiomyopathy (ARVC) and one patient suffered from muscular dystrophy of the limb-girdle type with cardiac involvement, secondary cardiomyopathy. Three patients presented with Brugada syndrome (BrS). One patient suffered from long-QT syndrome type 1 (LQTS1). Furthermore, two patients had congenital heart defects and one patient suffered from cardiac sarcoidosis (CS). There were no appropriate/inappropriate shocks with the WCD in this cohort. One patient had recurrent self-limiting sustained ventricular tachycardia during the wear time, but actively inhibited a shock and was hospitalized. The compliance rate in this cohort was 77.8% with a mean wear time of 45.3 ± 26.9 days with a mean follow-up time of 570 ± 734 days. 55.6% (10/18) of the patients received an ICD after WCD wear time.

**Conclusions:**

This retrospective study of patients with inherited and congenital heart disease shows that WCD use is not beneficial in the majority of patients with inherited and congenital heart disease.

## 1 Introduction

Inherited channelopathies and cardiomyopathies such as Long-QT syndrome (LQTS), Brugada syndrome (BrS), non-compaction cardiomyopathy (NCCM) and hypertrophic (non-) obstructive cardiomyopathy (HCM), as well as congenital heart defects are at high risk for ventricular tachyarrhythmias and sudden cardiac death (SCD) (1–4).

Implantable Cardioverter-Defibrillators (ICD) have been used for a long time in primary or secondary prevention of sudden cardiac death (SCD). However, ICDs might be related to several complications e.g., infection, lead failure or dysfunction in patients suffering from inherited cardiovascular diseases (5–7). Patients may refuse ICD implantation or there may be other reasons to postpone ICD implantation for the time being. Respectively, patients with suspected inherited heart disease might still have to undergo genetic testing or further risk stratification before ICD implantation. In addition, patients who already received an ICD might suffer from an infection and need a temporary ICD explantation and subsequently antibiotic therapy (8), or patients cannot receive an immediate ICD implantation because of a new medication with still insufficient dosage.

For these temporary high-risk situations, the wearable cardioverter-defibrillator (WCD) has been suggested and is used already in different patient groups and settings (9).

Studies regarding the use of the WCD in inherited and congenital heart disease are sparce and therefore currently not recommended by any international guideline. The 2022 ESC guideline for the management of patients with ventricular arrhythmias and the prevention of sudden death suggest WCD use in patients with a secondary prevention ICD indication, who are temporarily not candidates for ICD implantation and for patients in the early phase after myocardial infarction (4). Recently published data showed that the use of WCD could be beneficial in patients suffering from myocarditis and preserved systolic left ventricular ejection fraction (10).

Therefore, we sought to assess the utility of WCD in this cohort of patients with inherited and congenital heart disease.

## 2 Methods

### 2.1 Patient recruitment

In this multicenter, retrospective study, patients were recruited between April 2012 and December 2021. 1,675 patients with an indication and prescription of a WCD (LifeVest; ZOLL; Pittsburgh, PA) from nine hospitals (Bergmannsheil University Hospital, University Hospital Mannheim, Helios Clinic Krefeld, University Hospital St. Josef-Hospital Bochum, Klinikum St. Georg Leipzig, University Hospital Bonn, Clinic Saarbrücken, University Hospital Zurich, Frankfurt University Hospital) were included. The study was approved by the local ethics committee. All procedures, analyses and statistical evaluations were performed in accordance with guidelines and regulations of the institutional research committee and conform to the 1975 declaration of Helsinki.

### 2.2 Data collection from the wearable cardioverter defibrillator

Data from the WCD (wear time, wear days, arrhythmic events) was extracted from ZOLL LifeVest Network^TM^ and hospital patient records. The WCD was programmed as recently described (11). The ventricular fibrillation (VF) zone was programmed at a heart rate of 200–220 bpm with a response time of 25 s.

The events were classified and reviewed by at least two independent physicians. Ventricular tachycardia events were divided in sustained ventricular tachycardia (VT, lasting ≥30 s or causing hemodynamic compromise) and non-sustained VT (< 30 s). Shocks were sorted as appropriate shocks (the cause was VT or VF) or inappropriate shocks (the cause was a non-sustained VT or any other reason other than VT or VF).

A sufficient compliance was defined as an average of ≥20 wear hours per day.

### 2.3 Clinical data collection

All data was collected retrospectively using the electronic patient records and reports. For follow-up data, treating hospital archives were screened and treating physicians or patients were contacted.

Characterizing parameters of the patients such as gender, age, indication for WCD use and underlying heart condition and comorbidities were extracted.

Beside baseline characteristics at the initial hospital stay such as baseline left ventricular ejection fraction (LVEF), the New York Heart Association (NYHA) classification, ECG parameters (PR, QRS and QTc- interval) and medication at discharge, follow-up data was collected at the times of three-month (short-term) and six to twelve-month (long-term) follow-up. LVEF was measured by the biplane Simpson's method using echocardiography and/or cardiac magnetic resonance imaging (MRI). An improvement of LVEF was defined as ≥35% if baseline LVEF was below 35%. At the end of the WCD wear time, the reason for stopping WCD use was documented. Additionally, any cardiac implantable electronic devices (CIEDs) that might have been implanted after WCD use, were monitored for documented arrhythmic events if possible. During follow-up, data were collected on rehospitalizations or death.

### 2.4 Statistical analysis

Continuous variables were analyzed for normal distribution using the Shapiro-Wilk normality test. Data are presented as mean ± standard deviation if normally and as median with 25th and 75th percentile if non-normally distributed. Categorical variables are displayed as frequencies and percentages.

## 3 Results

### 3.1 Baseline characteristics

In the present cohort, we included 18 patients suffering from congenital and inherited heart disease. Nine patients (50%) were male with a mean age of 41.3 ± 16.4 years, Table 1.

**Table 1:** Baseline characteristics of the cohort.

Variables	All patients (*n* = 18)
Demographics	
Male, *n* (%)	9/18 (50)
Age, mean ± SD	41.3 ± 16.4
Comorbidities	
Coronary artery disease, *n* (%)	2/18 (11.1)
Myocardial infarction, *n* (%)	0/18 (0)
CABG, *n* (%)	0/18 (0)
Chronic obstructive pulmonary disease, *n* (%)	2/18 (11.1)
Chronic kidney disease/dialysis, *n* (%)	1/18 (5.6)
Atrial fibrillation or atrial flutter, *n* (%)	3/18 (16.7)
TIA/stroke, *n* (%)	1/18 (5.6)
Diabetes mellitus, *n* (%)	0/18 (0)
Smoker, *n* (%)	3/18 (16.7)
Hypertension, *n* (%)	2/18 (11.1)
Hyperlipidemia, *n* (%)	2/18 (11.1)
Overweight, *n* (%)	10/18 (55.6)
BMI kg/m^2^, mean ± SD	26.2 ± 5.9
Family history of cardiovascular disease, *n* (%)	4/18 (22.2)
Hospital side parameters	
Cardiogenic shock at diagnosis, *n* (%)	4/18 (22.2)
Pulmonary edema, *n* (%)	0/18 (0)
Days of hospitalization, mean ± SD	11.1 ± 9.7
Drug treatment	
ACE inhibitors, *n* (%)	9/18 (50)
ARNI, *n* (%)	0/18 (0)
Aldosterone antagonist, *n* (%)	3/18 (16.7)
ß blocker, *n* (%)	12/18 (66.7)
Amiodarone, *n* (%)	0/18 (0)
LVEF	
LVEF at index, mean ± SD (%)	48.7 ± 19.3
LVEF short-term, mean ± SD (%)	49.7 ± 16.6
LVEF long-term, mean ± SD (%)	55 ± 13.1

SD, Standard deviation; CABG, coronary artery bypass grafting; TIA, transient ischaemic attack; LVEF, left ventricular ejection fraction; BMI, body-mass-index; COPD, Chronic obstructive pulmonary disease; ACE, angiotensin-converting-enzyme; ARNI, angiotensin-converting enzyme inhibitors.

At the initial hospital stay, the mean LVEF of the whole cohort was 48.7 ± 19.3%, with a LVEF≤35% in four patients. Three of those had the diagnosis of non-compaction cardiomyopathy (NCCM) and one patient suffered from muscular dystrophy of the limb-girdle type with cardiac involvement. None of these patients had an improvement of their LVEF over the first 12-months, Table 1. Detailed baseline characteristics are shown in Table 1.

### 3.2 Wearable cardioverter defibrillator data and indications for WCD use

Primary preventive protection by WCD was recommended in eleven patients, seven patients received the WCD as secondary prevention due to ventricular tachyarrhythmia prior to WCD use.

The average wear hours of the WCD in this cohort were 20 ± 6.4 h per day, with a compliance rate of 77.8%. The mean wear time of the WCD was 45.3 ± 26.9 days with only one patient (patient nr. 17) wearing the WCD for more than 90 days, Table 2.

**Table 2:** WCD data.

Variables	All patients (*n* = 18)
Recorded WCD data	
Wear days, mean ± SD	45.3 ± 26.9
Average wear hours, mean ± SD	20 ± 6.4
More than 90 wear days, *n* (%)	1/18 (5.5)
Compliance (>20 h per day of wear time), *n* (%)	14/18 (77.8)
Arrhythmic episodes during WCD	
Sustained ventricular tachycardia, *n* (%)	1/18 (5.5)
Ventricular fibrillation, *n* (%)	0/18 (0)
Non-sustained ventricular tachycardia, *n* (%)	0/18 (0)
WCD shocks	
Appropriate, *n* (%)	0/18 (0)
Inappropriate, *n* (%)	0/18 (0)
Inhibition of shocks by patient, *n* (%)	5/18 (27.8)
Recorded atrial fibrillation or atrial flutter by WCD, *n* (%)	1/18 (5.5)
Recorded AV-block or asystole by WCD, *n* (%)	0/18 (0)
Reason for stopping WCD	
Improved LVEF, *n* (%)	1/18 (5.5)
Definite ICD implantation, *n* (%)	10/18 (55.6)
Incompliance, *n* (%)	2/18 (11.1)
Death, *n* (%)	1/18 (5.5)
Unknown, *n* (%)	2/18 (11.1)
Low arrhythmic risk with normal LVEF, *n* (%)	2/18 (11.1)

SD, Standard deviation; LVEF, left ventricular ejection fraction

27.8% of the cohort actively inhibited shocks due to false alarms, Table 2. Artefacts, including those caused by movement, led to warning signals that can be followed by shocks if not inhibited by the patient. All of these false alarms were inhibited actively by the patients with the response-button. All other artefacts or supraventricular tachycardias were correctly detected by the WCD and did not result in a warning signal, nor an inappropriate WCD shock. No appropriate WCD shocks were documented in this cohort during follow-up.

#### 3.2.1 Hypertrophic cardiomyopathy

In our cohort, four patients suffered from hypertrophic cardiomyopathy (HCM), one of them with an obstructed left ventricular outflow tract (patient nr. 2) and three patients without (patients nr. 1, 3 and 4). Three out of four patients used the WCD as secondary prevention due to prior sustained VT/VF, their mean LVEF was 59.1%. Patient nr. 1 had an infection of her ICD and was bridged during antibiotic therapy with the WCD. Patient nr. 2 received an event recorder two years prior, due to recurrent presyncope. He was then admitted to the hospital in 2015 due to a tumor excision and the event recorder showed multiple nsVTs, which led to an ICD indication. The tumor excision delayed ICD implantation by a few weeks, which was bridged by the WCD.

Patient nr. 3 and 4 were newly diagnosed with HCM and received the WCD after survived SCD in times of further diagnostic measures. Individual patient data regarding definite ICD implantation and follow-up is provided in Table 3.

**Table 3:** Patient information.

Patient number (gender/age)	Disease	Indication for WCD	VT/VF in WCD	ICD-implantation	ICD shock	VT/VF prior to WCD use	EF baseline (%)
1 (F/16)	HNOCM	After ICD infection WCD as bridge	No	Yes	No	No	55
2 (F/60)	HOCM	S/P implanted event recorder following multiple syncopes: detection of nsVTs and ICD-indication.	No	Yes	No	Yes	60
		Now: tumor excision → temporarily ICD-implantation not possible					
3 (M/49)	HNOCM	CPR (VF)	No	Patient denied		Yes	56.4
4 (F/13)	HNOCM (no genetic mutation found)	Secondary prevention	No	Yes	No	Yes	61
5 (F/49)	ARVC	Diagnostic	No	Yes	No	No	61.5
6 (F/46)	ARVC	CPR (VF)	No	Yes	Yes (inappropriate shock)	Yes	74.4
7 (M/76)	Brugada syndrome (variant of the SCN5A gene)	WCD as bridge for patient decision about ICD; S/P syncope and inducible VT and VF in EPS in 2007	No	Yes	No	No	Unknown
8 (M/61)	Brugada syndrome	Syncope; WCD as bridge until ICD	No	Yes	No	No	60
9 (M/32)	Brugada syndrome	Inducible VF in EPS	No	Yes	Yes (inappropriate shock)	No	56.9
10 (F/40)	Long-QT-syndrome (point mutation in the KCNQ1 gene)	New ADHD medication	No	No		No	60
11 (M/36)	Non-compaction CM	EF ≤35%	No	No		No	15.1
12 (M/50)	Non-compaction CM	Family history of SCD	No	No		No	39.4
13 (F/17)	Non-compaction CM	EF ≤35%	No	Yes	Yes (VT/VF)	No	20
14 (M/51)	Non-compaction CM	EF ≤35% & secondary prevention	No	Yes	No	Yes	27
15 (F/42)	Muscular dystrophy of the limb-girdle type with cardiac involvement	VF after ICD-explantation & EF ≤35%	Yes	Died before implantation		Yes	13
16 (M/38)	Transposition of the great arteries	Suspected shunt defect and reprogramming of the CRT-D	No	N/A	No	No	60
17 (F/28)	Pulmonary atresia with intact ventricular septum	Premature ventricular contractions and short ventricular salvo	No	No		Yes	60
18 (M/39)	Cardiac sarcoidosis	unknown	No	Unknown		No	Unknown

#### 3.2.2 Non-compaction cardiomyopathy

Another four patients suffered from non-compaction cardiomyopathy (NCCM), patients nr. 11, 12, 13 and 14. All four patients had severe LV dysfunction, with an LVEF≤35% in three patients and an LVEF≤40% in one patient with a mean LVEF of 25.4% in this population. Only patient nr. 14 had a prior sustained ventricular tachyarrhythmias.

#### 3.2.3 Arrhythmogenic right ventricular cardiomyopathy

Two patients (nr. 5 and 6) were diagnosed with arrhythmogenic right ventricular cardiomyopathy (ARVC) and subsequently wore the WCD. One patient (patient nr. 5) had further diagnostic measures and wore the WCD as a bridging option, and one patient (patient nr. 6), had cardiac arrest and was incompliant, but declined the use of the WCD after only two hours due to discomfort.

#### 3.2.4 Brugada syndrome

Three patients presented with Brugada syndrome (BrS) (patients nr. 7, 8 and 9):

All three patients used the WCD as primary prevention for SCD. Patient nr. 7 had a previous dysfunction of the ICD but was not sure whether he wanted another ICD and the WCD was used for bridging during this decision period. This patient previously had a syncope, and VF was induced during the electrophysiological study in 2007. Patients nr. 8 and 9 both received their WCD, as diagnostics for BrS and differential diagnosis were still going on. Patient nr. 8 presented with a syncope and patient nr. 9 with an inducible VF in EPS. Besides 19 false alarms in patient nr. 8, there were no conspicuities during the WCD wear time.

#### 3.2.5 Long-QT syndrome

Patient nr. 10 suffered from long-QT syndrome type 1 (LQTS1). The patient received the WCD as a primary prevention method for SCD and monitoring option while being dosed up on methylphenidate for her previously diagnosed “Attention Deficit Hyperactivity Disorder” (ADHD). This patient did not have an ICD indication and did not receive an ICD after the WCD wear time, but physicians worried about possible QT-time prolongation due to the new drug, which can prolong the QTc interval, and prescribed the WCD as a monitoring device. After titration of methylphenidate the QTc interval was 470 ms at maximum with no clinical symptoms. The WCD and monitoring were terminated without any further ECG abnormalities after a wear time of 60 days.

#### 3.2.6 Congenital heart defect

Two patients (patients nr. 16 and 17) had congenital heart defects. Patient nr. 16 had a corrected transposition of the great arteries, had a suspected shunt defect and was at the hospital for reprogramming of his CRT-D and deactivating his tachyarrhythmia absoluta. Patient nr. 17 suffered from pulmonary atresia with an intact ventricular septum and used the WCD as secondary prevention due to prior sustained VT/VF.

#### 3.2.7 Cardiac sarcoidosis

Patient nr. 18 was diagnosed with cardiac sarcoidosis (CS) but unfortunately stopped the WCD treatment after only five hours due to incompliance and concerns regarding the device.

#### 3.2.8 Secondary cardiomyopathy

Lastly, patient nr. 15 suffered from muscular dystrophy of the limb-girdle type with cardiac involvement, secondary cardiomyopathy, Table 3.

The patient received the WCD as a bridging therapy after ICD-system explantation due to device malfunction and received a temporary CRT-P due to no intrinsic rhythm. After explantation of the ICD, the patient suffered from an episode of ventricular fibrillation in the hospital, whose termination was unsuccessful by multiple defibrillations and amiodarone administrations and was ultimately achieved by lowering the heart rate due to overstimulation with the temporary pacemaker. This arrhythmia was followed by episodes of sustained VTs. The WCD was used as a bridging therapy. There were no shocks delivered by the WCD. Unfortunately, this patient was admitted one month later with recurrent self-limiting sustained VTs, also documented by the WCD. The patient died shortly after admission due to cardiogenic shock, despite extensive attempts of resuscitation. Data regarding definite ICD implantation and follow-up is provided in Table 4.

**Table 4:** ICD-implantation and follow up.

Variables	All patients (*n* = 18)
Cardiac implantable electronic devices	
ICD implantation, *n* (%)	10/18 (55.6)
Appropriate ICD shocks, *n* (%)	1/10 (10)
Inappropriate ICD shocks, *n* (%)	2/10 (20)
Arrhythmic episodes post-ICD	
Sustained ventricular tachycardia, *n* (%)	1/10 (10)
Ventricular fibrillation, *n* (%)	1/10 (10)
Others, *n* (%)	2/10 (20)
Follow up data	
Death during follow-up, *n* (%)	1/18 (5.5)
Hospitalization, *n* (%)	6/18 (33)
Cardiovascular cause of rehospitalization, *n* (%)	3/6 (50)
Ventricular tachycardia/ventricular fibrillation as cause of rehospitalization, *n* (%)	2/6 (33.3)
Congestive heart failure as cause of rehospitalization, *n* (%)	1/6 (16.7)
Non-cardiovascular cause for rehospitalization, *n* (%)	3/6 (50)

The main reason for stopping WCD use was definite ICD implantation in 10 out of 18 patients (55.6%). Patient nr. 12 had an LVEF improvement large enough to discontinue WCD use. Two patients (patient nr. 3 and 18) stopped WCD treatment due to incompliance and two patients stopped WCD use due to a normal LVEF from the start and no arrhythmias during the wear time (patient nr. 10 and 16), Table 2. Unfortunately, in two patients the reason for stopping the WCD use was unknown.

### 3.3 ICD implantation and follow-up

The mean follow-up time of the cohort was 570 ± 734 days. 55.6% (10/18) of the patients received an ICD after WCD wear time as shown in Tables 3, 4. Three of these patients received an appropriate (*n* = 1) /inappropriate (*n* = 2) shock during follow-up, Tables 3, 4. Patient nr. 13 with NCCM suffered from sustained VT7VF resulting in an appropriate shock 126 days after stopping the WCD treatment and during the use of the ICD. This patient had not had any form of ventricular arrhythmia before and received the WCD as primary prevention due to an LVEF of 20% at baseline. Due to persistent LVEF≤35% an ICD was implanted. The other two patients received an inappropriate shock (patient nr. 9 and 14), Tables 3, 4.

Six patients were hospitalized, three out of six due to a cardiovascular cause. Two of these patients were hospitalized due to sustained VT/VF and one patient suffered from congestive heart failure. The remaining three patients had a non-cardiovascular cause of hospitalization, Table 4.

## 4 Discussion

The European Society of Cardiology (ESC) recommends WCD use in adult patients with a secondary prevention ICD indication, who temporarily cannot be implanted with an ICD (class IIa recommendation) (4). The American Heart Association (AHA) states in the 2016 WCD Science Advisory that the use of the WCD may be reasonable when there is concern about an increased risk of SCD that may resolve over time (class IIb recommendation) and recommends the use of WCD *in situ*ations associated with increased risk of death in which ICDs have been shown to reduce SCD but not overall survival (class IIb recommendation) (12).

In our multicenter WCD-registry with 1,675 patients, we identified 18 patients with an inherited or congenital heart disease. 61.1% received the WCD as primary prevention and in 38.9% the WCD was recommended for secondary prevention due to sustained ventricular tachyarrhythmia prior to WCD use.

None of these patients received an appropriate WCD shock. Only one patient (patient nr. 15) had self-limiting sustained VTs which were correctly identified by the WCD but a shock was avoided by the patient. In five patients there were warning signals due to artefacts, but WCD shock delivery was stopped with the response button by the patients.

This shock rate of 0% was previously described in other studies about inherited and congenital heart diseases. E.g., Sarubbi et al. described eight patients with congenital heart disease, also without WCD shocks (13).

Nevertheless, Rao et al. described a cohort of 43 patients with congenital and 119 patients with inherited heart disease from the USA with two appropriate shocks and four inappropriate shocks, resulting in an event rate of 27 appropriate shocks per 100 patient-years. All shocks were received by patients with an inherited heart disease (14).

Likewise, Owen et al. described a cohort of nine LQTS patients with one appropriate shock after two days of wear time in a patient after multiple syncopal episodes and seizures (15).

Spar et al. described a cohort in pediatric patients with 167 patients with cardiomyopathy, 90 patients with congenital heart disease, 47 patients with channelopathy and 36 patients with cardiac arrest without diagnosis, where two appropriate shocks were documented, both in the congenital heart disease group (16).

Therefore, we may argue that the WCD is not beneficial for all patient groups with inherited and congenital heart disease. Even though there still is a lack of data for the use of WCD in these patients, in the published studies there are often no shocks or a low shock rate, similar to our study (13, 14, 16, 17). Most appropriate shocks that are documented occurred in patients with inherited heart disease.

However, as Rao et al. (14) shows, there might also be a considerable inappropriate shock rate, with an event rate of 63 inappropriate shocks per 100 patient-years in these patients. In our study, there were no inappropriate shocks with the WCD, but two inappropriate shocks during follow-up with the ICD (patients nr. 9 and 16), leading to an inappropriate shock rate of 20%. Of note, 27.8% of patients avoided an inappropriate WCD shock after being alarmed by the WCD by pressing the response-button.

In this cohort, there are many different individual reasons and clinical scenarios for WCD use.

The LQTS1 patient (patient nr. 10), received the WCD as a monitoring option while being dosed up on methylphenidate for their previously diagnosed ADHD. The 2022 ESC guidelines for management of patients with ventricular arrhythmias and prevention of SCD (4) states that drugs, that are known to prolong of the QTc time should be avoided for the prevention of SCD in the LGTS. In the case of our patient, the LQTS had been diagnosed a few years prior the current starting of medication with methylphenidate. After an initial prolongation of the QTc up to 520 ms years ago, LQTS was diagnosed, and the medication stopped. Now, after a few years, the patient again needed to be dosed up with methylphenidate and received the WCD as a way of bridging this time to an established dose and high risk for SCD.

Long-QT syndrome has an absolute SCD risk of 4.9%–13% (18) and has a heightened risk for Torsade de Pointes tachycardia (2). In 13% of patients, a sudden cardiac arrhythmia or death occurs before age 40. The magnitude of the QT interval prolongation remains the most powerful risk factor for SCD with a QTc>500 ms as a described predictor for life-threatening events (19). In patient nr. 10, after a wear time of 60 days and a stable maximum QTc time of 470 ms with no clinical symptoms, the WCD and monitoring were terminated, without any further ECG abnormalities. No permanent ICD was implanted, and the treatment of this patient was continued with betablockers.

Another example of individual indications and benefits from the WCD are the two patients (nr. 5 and 6) suffering from ARVC. One patient (patient nr. 5) had further diagnostic tests for ARVC and wore the WCD as a bridging option, and the other patient (patient nr. 6) had cardiac arrest but declined the use of the WCD after only two hours due to discomfort. This patient was implanted with an ICD, with whom he experienced an inappropriate shock during follow-up. Risk factors described in the literature for SCD in ARVC patients, were not met in patient nr. 5 (20–24). In patient nr. 6, the main risk factor was the previous cardiac arrest.

Consequently, these were two patients with very different presentations of ARVC. On the one hand, patient nr. 5 had no prior arrhythmic events and was implanted with a primary prevention ICD without any ICD-related complications, the WCD solely being the bridge until the diagnostic tests were completed and the diagnosis of ARVC confirmed. She wore the WCD as a safety measure until ICD implantation but had no apparent benefit from it.

Patient nr. 6 on the other hand, was at a very high risk for SCD, because of the previous cardiac arrest. She declined WCD use after two hours and was implanted with an ICD but had an inappropriate shock shortly after. This patient might have benefited from the WCD. Moreover, with the WCD she could have manually aborted the inappropriate shock with the response button, which may have a led to appropriate measures to avoid an inappropriate shock with the definite ICD.

A pooled annualized incidence rate of both aborted SCD and SCD without ICD treatment by Agbaedeng et al., describes a rate of 12.66 per 1,000 patients with ARVC. Appropriate ICD shocks are described at an annual rate of 84.7 per 1,000 patients (21).

Kutyifa et al. describes a congenital/inherited subgroup of their prospective study with WCD use which also included ARVC patients. Patients with ischemic and congenital/inherited heart disease were shown to have significantly higher probabilities of VT/VF than those with nonischemic cardiomyopathy with a rate of sustained VT of 3% after 3 months (25).

In this study, we also looked at patients with non-compaction cardiomyopathy (NCCM) which presents itself with an alteration of trabeculation (26–27). VTs are reported in 38%–47% of adult patients with major risk factors, these being syncope, low LVEF, previous VT/VF and family history of SCD (26).

Patient nr. 13 did not have multiple risk factors for ventricular tachyarrhythmias, except a low EF of 20% which improved to 32% over the first year. This patient received an appropriate ICD shock 126 days after ICD-implantation, due to recorded VT and VF. This may speculate, that the risk for SCD might also be high in some patients with NCCM, with their only risk factor being a low LVEF, where an ICD implantation should be considered early.

Patient nr. 15 with muscular dystrophy of the limb-girdle type with cardiac involvement and secondary cardiomyopathy, received their WCD as a bridge after ICD-system explantation. This patient had a history of VT/VF and suffered from ventricular fibrillation after explantation and from self-limiting VTs while wearing the WCD. After hospitalization, the patient sadly died shortly after with cardiogenic shock. To our knowledge, there is no data available on the rate of annual SCD in patients with muscular dystrophy of the limb-girdle type with cardiac involvement. These cases are associated with an alteration of different ion channel currents, which may enhance the risk of SCD (28). It is known that there is a heightened risk of atrial arrhythmias, conduction disease, bradycardia, ventricular arrhythmias, and sudden cardiac death, with ventricular arrhythmias and conduction disease often occurring after the development of a dilated cardiomyopathy (29–31). Therefore, ICD indication is usually guided by guidelines for non-ischemic cardiomyopathy (NICM) patients (29). Risk factors for SCD in the muscular dystrophy group are, nsVT, male sex and LVEF<45% (29). Patient nr. 15 had multiple risk factors for SCD e.g., an EF of 13% and benefited from the WCD in that she was alarmed for sustained VT and went to the hospital.

Overall compliance in our cohort was 77.8%, with an average wear time of 20 ± 6.4 h. Nevertheless, when removing patients nr. 18 and 6 due to immediate incompliance, compliance rate was at 87.5% with an average wear time of 22.1 ± 2.1 h. This condensed group wore the WCD 51 ± 22.7 days on average.

Our compliance rate can be compared to previously published studies, with Spar et al. (16) reporting a median wear time of 20.6 h per day, Sarubbi et al. (13) a wear time of 21.2 ± 1 h/d, Rao at al. (14). a compliance for daily use of 91% and Kutyifa et al. (25) a median daily use of 22.5 h of the whole cohort.

Olic et al. also reports patients with inherited and congenital heart disease stopping WCD use due to incompliance and discomfort, but that may be influenced by the fact that their patients were pregnant at the time and consequently of younger age (32). In our previous study regarding age differences in WCD use, the wear hours in younger patients (age <45 years) tended to be lower (20.11 ± 4.88 h) and predictors for worse compliance were younger age and non-ischemic cardiomyopathy (33). Therefore, we may argue, that the present cohort with an average age of 41 years may be less compliant to WCD use due to inherited and congenital heart diseases mainly being present in a younger age group.

## 5 Study limitations

Due to the limited size of this cohort, the retrospective data collection and the heterogeneity of the underlying disease, this study has limitations regarding direct applicability to clinical scenarios. Nevertheless, the aim of this study was to present more data on these rare indications and diseases for WCD in a systematic fashion.

## 6 Conclusion

This retrospective study of patients with inherited and congenital heart disease shows, that WCD use is not beneficial in the majority of patients with inherited and congenital heart disease. The appropriate WCD shock rate seems to be relatively low, especially in patients with congenital heart disease. Nevertheless, indications and reasons in this patient group varied individually and the WCD might be a good way to bridge some patients at high risk for SCD until definite ICD implantation, completion of further work-up and optimal drug therapy.

## Data Availability

The raw data supporting the conclusions of this article will be made available by the authors, without undue reservation.
